# Glycolytic Enzyme HK2 Phosphorylates nSMase1 to Promote Astrocytic Exosomes Biogenesis Contributing to Acute Ischemic Stroke Injury

**DOI:** 10.1002/advs.202501894

**Published:** 2025-07-28

**Authors:** Chen Chen, YuWei Zhou, LongXiang Sheng, FangYing Mai, YuXuan Ding, ShiRan Hong, JiaXin Wu, JiaKai Pi, GuangMei Yan, YiJun Huang, JingJing Duan, Xuefeng Hua, Wei Yin

**Affiliations:** ^1^ Department of Biochemistry and Molecular Biology Zhongshan School of Medicine Sun Yat‐Sen University Guangzhou 510080 China; ^2^ Department of Pharmacology Zhongshan School of Medicine Sun Yat‐Sen University Guangzhou 510080 China; ^3^ Department of Laboratory Medicine the Third Affiliated Hospital of Sun Yat‐sen University Guangzhou 510630 China; ^4^ The First School of Clinical Medicine Southern Medical University Guangzhou 510515 China; ^5^ The Department of Human Anatomy Zhongshan School of Medicine Sun Yat‐Sen University Guangzhou 510080 China; ^6^ Department of HBP Surgery Guangzhou First People's Hospital School of Medicine South China University of Technology Guangzhou 510180 China

**Keywords:** acute ischemic stroke, astrocyte, exosome, hexokinase 2

## Abstract

Stroke represents a significant threat to public health, with insufficient understanding of its pathological mechanisms hindering therapeutic advancements. Exosomes are implicated in both the injury and recovery processes of ischemic stroke. While enhanced glycolysis is linked to exosome biogenesis, its regulatory mechanisms remain largely unexplored. Here, an increase in the number of exosomes within the cerebrospinal fluid (CSF) and plasma in acute ischemic stroke is observed. Hypoxia selectively enhanced the exosomes biogenesis in astrocytes. Through RNAi screening, we identified the glycolytic enzyme hexokinase 2 (HK2) as a key promoter of hypoxia‐induced exosomes. HK2 facilitates the formation of intraluminal vesicles (ILVs), the precursors of exosomes, without affecting their degradation in lysosomes. HK2 directly phosphorylates neutral sphingomyelinase 1 (nSMase1), a critical enzyme involved in the lipid biogenesis pathway of exosomes. Moreover, hypoxia‐induced astrocytic exosomes disrupt cerebrovascular endothelial tight junction proteins. Astrocyte‐specific knockdown of HK2 significantly reduces exosomes release and alleviates brain injury caused by middle cerebral artery occlusion (MCAO). Notably, MCAO markedly increased the phosphorylation of nSMase1, which is effectively abolished by astrocytic HK2 knockdown. In conclusion, this study reveals a non‐metabolic role of HK2 in exosomes biogenesis via its protein kinase activity, offering a potential therapeutic target for stroke.

## Graphical abstract

This study reveals that the glycolytic enzyme HK2 mediates hypoxia‐induced astrocytic exosome biogenesis by directly phosphorylating nSMase1. These astrocytic exosomes impair brain endothelial tight junctions. Astrocyte‐specific knockdown of HK2 reduces exosome release and alleviates brain injury after MCAO. These findings highlight a nonmetabolic protein kinase role for HK2 in exosome production and establish HK2 as a potential therapeutic target for stroke.

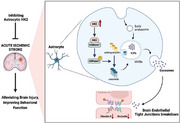

## 1 Introduction

Exosomes, a subclass of extracellular vesicles (EVs) ranging in diameter from 40 to 160 nm,^[^ ^1^ ^]^ serve as crucial mediators of intercellular communication. Accumulating evidence indicates that exosomes also play a key role in the onset, progression, and repair of both acute and chronic neurological diseases, including stroke.^[^ ^1^, ^2^ ^]^ The intracellular biogenesis of exosomes is primarily regulated by the endosomal sorting complex required for transport (ESCRT) system and the ceramide‐mediated lipid pathway. The ESCRT pathway is a highly conserved vesicle budding mechanism in eukaryotes that controls the formation of intracellular vesicles, phagocytosis, and virus infection of host cells.^[^ ^3^, ^4^, ^5^ ^]^ In contrast, the lipid pathway is a stress‐induced mechanism activated in response to inflammatory and oxidative stress.^[^ ^6^ ^]^ Neutral sphingomyelinase (nSMase), a key enzyme in the lipid pathway, catalyzes the conversion of sphingomyelin to ceramide, inducing membrane curvature and promoting ILVs formation.^[^ ^7^, ^8^ ^]^ Enhanced glycolysis has been linked with the generation of exosomes with unclear mechanism.^[^ ^9^ ^]^ Tumor cells exhibit a distinct high glycolytic metabolic pattern and secrete significantly much more exosomes than normal cells.^[^ ^4^, ^10^ ^]^ Hypoxia also elevates exosome levels in prostate^[^ ^11^ ^]^ and breast cancer cells,^[^ ^12^ ^]^ while glycolysis inhibitors Shikonin significantly suppress the release of exosomes by targeting the glycolytic enzyme pyruvate kinase type M2 (PKM2).^[^ ^13^ ^]^ Moreover, glycolytic enzymes were found colocalized in exosomes or extracellular vesicles,^[^ ^14^ ^]^ suggesting their potential involvement in the generation or secretion of exosomes.

Stroke is one of the most prevalent cardiovascular and neurological disorders, ranking as the second leading cause of death globally and the primary contributor to disability and dementia.^[^ ^15^ ^]^ Despite great advances in ischemic stroke prevention and treatment in recent years,^[^ ^16^ ^]^ a comprehensive understanding of the underlying molecular mechanisms of ischemic stroke is essential for the development of novel therapeutic agents.^[^ ^17^ ^]^ During cerebral ischemia, the reduction of oxygen supply leads to an increase in glycolysis levels,^[^ ^18^ ^]^ suggesting that the glycolytic pathway is implicated in the process of brain injury. However, the precise mechanisms by which glycolytic enzymes contribute to brain injury remain poorly understood.

Here, we discovered that acute ischemic stroke (AIS) leads to a significant increase in exosome production, predominantly originating from astrocytes. Transcriptomic profiling reveals that hypoxia markedly activates the glycolytic pathway in astrocytes. Through RNAi screening, we further identified HK2, a rate‐limit enzyme catalyzing the first step of the glycolysis pathway, as a crucial regulator for the exosomes production in astrocytes under hypoxic conditions. HK2 was found to directly interact with nSMase1, phosphorylating it to facilitate exosomes generation in hypoxic astrocytes. Moreover, exosomes derived from hypoxic astrocytes are shown to disrupt endothelial tight junction proteins. Selective knockdown of HK2 in astrocytes reduces exosomes release and alleviates acute ischemic brain injury in mice. Collectively, our results demonstrate that HK2 acts as a protein kinase to mediate exosome biogenesis in astrocytes following stroke, thereby highlighting its non‐metabolic mechanism and pivotal role in brain injury.

## 2 Result

### 2.1 The Number of Exosomes in the CSF and Plasma is Increased in Acute Ischemic Stroke

**Figure 1:**
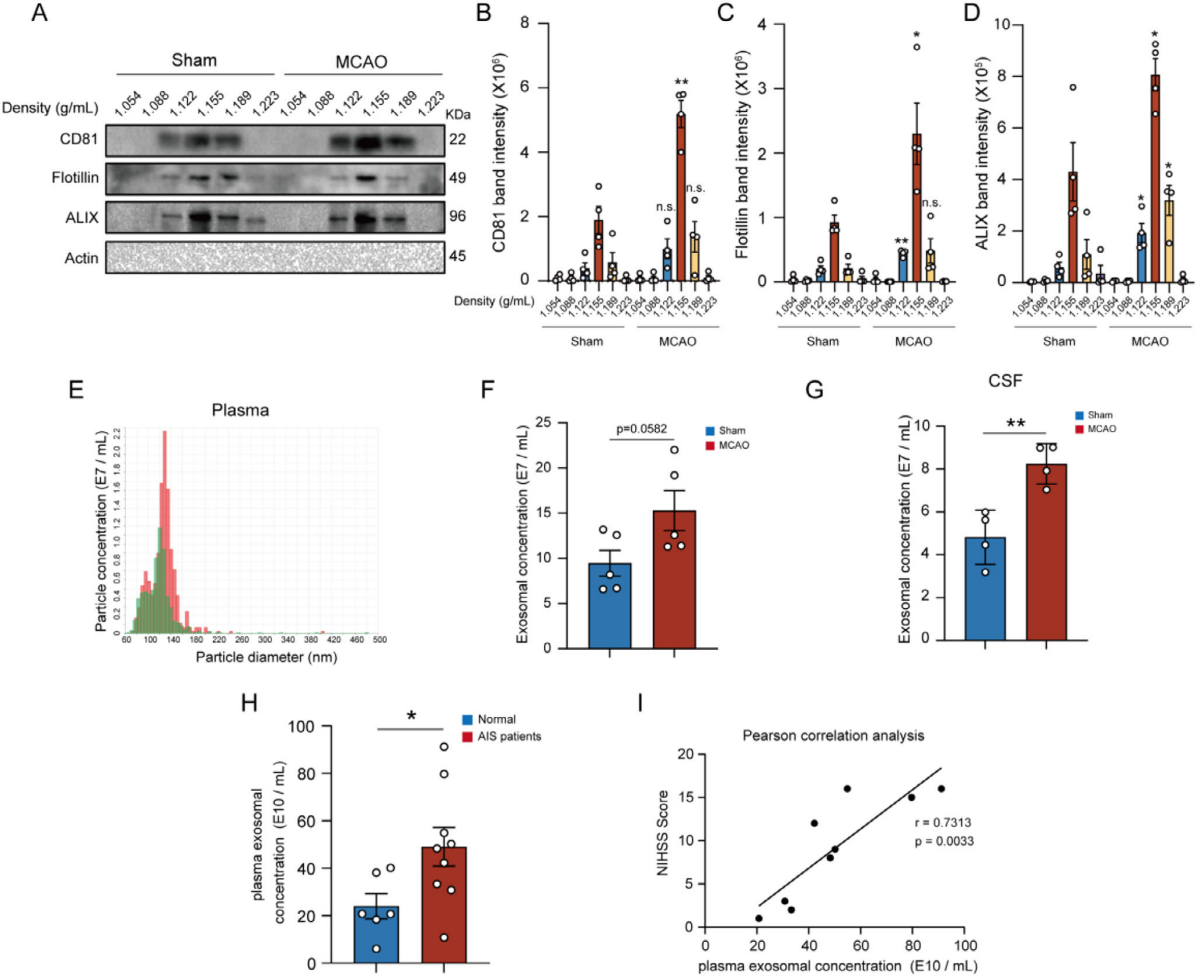
The number of exosomes in the CSF and plasma is increased following acute ischemic stroke. A) Exosomes were isolated from the same volume of plasma of sham and MCAO mice, and analyzed by Western blot using the exosome markers CD81, Flotillin, and ALIX, with cytoplasmic Actin serving as a negative control. B–D) Quantification of CD81 B), Flotillin C) and ALIX D) band intensity in (A). E) qNano particle diameter analysis of exosomes isolated from the plasma of Sham/MCAO mice. F–G) Particle concentrations of exosomes isolated from plasma F) and CSF G) of sham and MCAO mice. H) Particle concentrations of exosomes isolated from plasma of AIS patients and normal individuals. I) Pearson correlation analysis between NIHSS score with concentrations of exosomes. Data were presented with Mean ± SEM. Statistical analysis was performed by an unpaired Student's *t*‐test. n.s., no significance. ^*^ *p* < 0.05; ^**^ *p* < 0.01.

To investigate the impact of AIS on exosomes production, mice underwent MCAO surgery to simulate 1 h of ischemia, followed by 23 h of reperfusion after occlusion removal. Plasma‐derived exosomes were then isolated through density gradient ultracentrifugation. The densities of these isolated exosomes ranged from 1.122 to 1.223 g mL^−1^, aligning with the established exosomal density range of ≈1.13–1.19 g mL^−1^ (**Figure** **1**A). These exosomes were further verified to contain canonical exosomal markers CD81, Flotillin, and apoptosis‐linked gene 2‐interacting protein X (ALIX), while being free from cytoplasmic contaminants such as Actin (Figure 1A). Moreover, the protein levels of these markers were significantly increased following MACO surgery (Figure 1B–D). Particle tracking analysis by qNano further revealed that MCAO induced a slight but no significant increase in the number of isolated exosomes from plasma, with the particle diameters ranging approximately from 60 to 140 nm (Figure 1E,F). Additionally, the number of exosomes in the CSF of MCAO mice was significantly higher compared to that of sham‐operated group (Figure 1G). We further isolated exosomes from the peripheral blood samples of the ischemic stroke patients and healthy individuals, and found that the number of exosomes from AIS patients was remarkably higher than that of healthy individuals (Figure 1H). Moreover, the plasma exosomal concentration was positively correlated with the National Institutes of Health Stroke Scale (NIHSS) score of neurological function (Figure 1I). These findings demonstrated that AIS induces an elevation of exosomes in the CSF and plasma, suggesting their potential involvement in stroke‐related pathophysiology.

### 2.2 Hypoxia Selectively Promotes the Biogenesis of Exosomes in Astrocytes

Exosomes originate from the membrane lumen within early endosomal pathway and the plasma membrane recycling process.^[^ ^19^ ^]^ Early endosomes progressively mature into late endosomes within the cytoplasm, during which their limiting membranes invaginate to form the exosomal precursors of ILVs.^[^ ^20^ ^]^ Late endosomes containing multiple ILVs, also known as multivesicular bodies (MVBs), either fuse with lysosomes for content degradation or with the plasma membrane to release ILVs into the extracellular space as exosomes.^[^ ^21^ ^]^ It has been shown that exosomes isolated from mouse brain tissue after transient MCAO surgery are predominantly derived from astrocytes.^[^ ^22^ ^]^ We further analyzed single‐cell RNA sequencing data (GSE174574) from mouse brains after MCAO^[^ ^23^ ^]^ (Figure S1A,B, Supporting Information), and found significant enrichment of Gene Ontology (GO) terms related to extracellular vesicles and exosomes in astrocytes (Figure S1C, Supporting Information). Moreover, canonical exosome markers in astrocytes were significantly upregulated compared to other cell populations (Figure S1D, Supporting Information).

**Figure 2:**
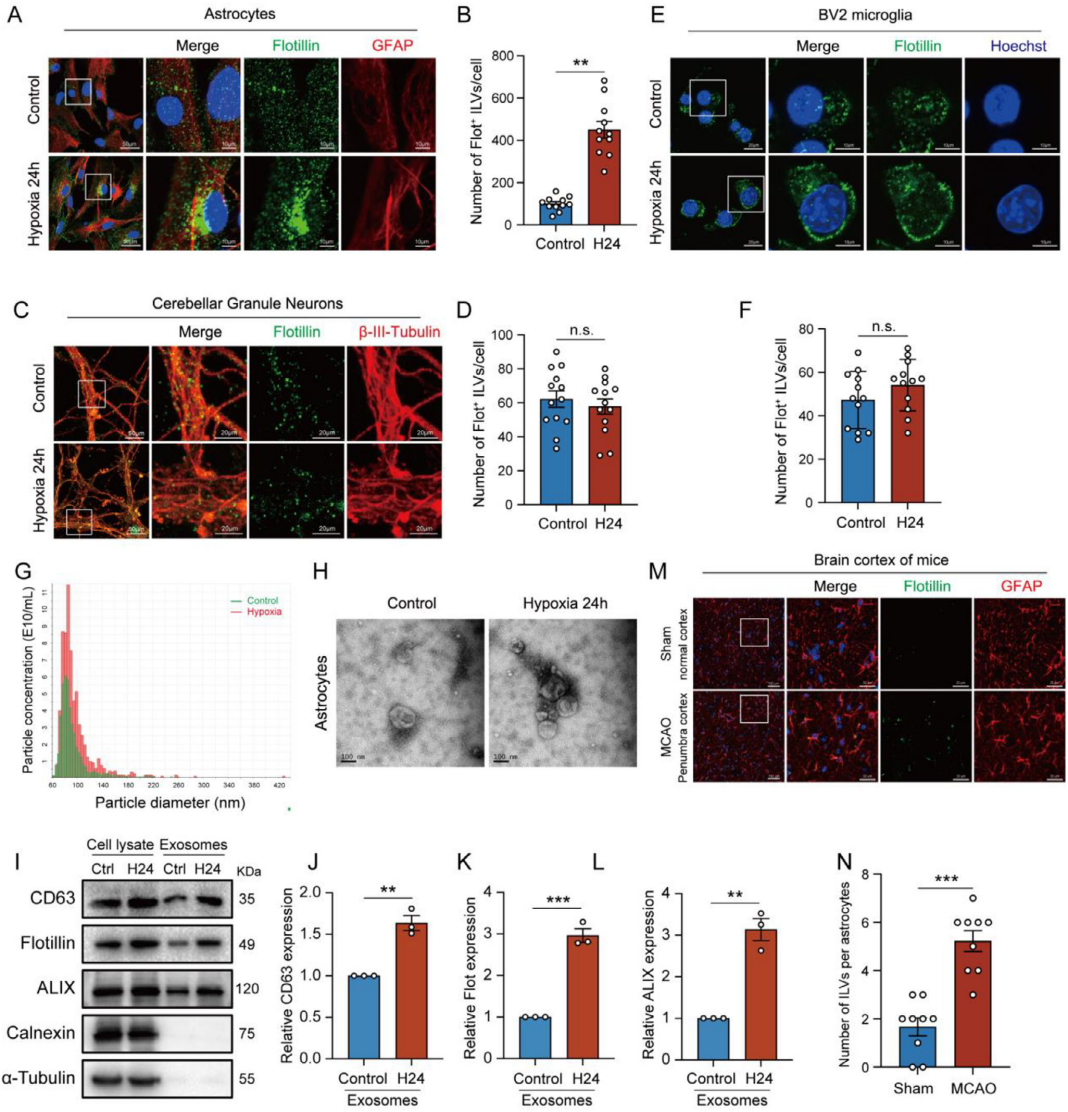
Hypoxia promotes the biogenesis of exosomes in astrocytes selectively. A–F) Representative immunofluorescence images and quantity statistics of Flot^+^ ILVs (green) in hypoxic or normoxic primary astrocytes (red), bar = 50 µm (left), bar = 10 µm (right) A,B), primary cerebellar granule neurons (red), bar = 50 µm (left), bar = 20 µm (right) C,D) or BV2 microglia, bar = 20 µm (left), bar = 10 µm (right) E,F) respectively. G) Particle size distribution of exosomes isolated from astrocytes under normoxic or hypoxic conditions, measured by IZON qNano particle analysis. H) TEM analysis of the morphology of exosomes released by astrocytes under normoxia or hypoxia. I) Western blot analysis of exosomal marker proteins CD63, Flotillin, and ALIX, along with negative markers Calnexin and α‐Tubulin, in cell lysates and exosome lysates from equal amounts of astrocytes treated with or without hypoxia. J–L) Quantification of CD63 J), Flotillin K) and ALIX L) protein levels normalized to Control group in (I). M) Representative immunofluorescence images of Flot^+^ ILVs (green) in GFAP‐labeled astrocytes (red) from the normal or penumbra cortex of MCAO mice. N) Quantity statistics of Flot^+^ ILVs in (J). Data were presented with Mean ± SEM. H24 represents hypoxia treatment with 1% O_2_ for 24 h. Statistical analysis was performed by unpaired Student's *t*‐test. n.s., no significance. ^**^ *p* < 0.01; ^***^ *p* < 0.001.

To further explore the cellular sources of exosomes released during the ischemic stroke, the quantity of ILVs was evaluated in primary mouse cerebellar granule neurons, primary mouse astrocytes and BV2 microglial cells following 24 h of 1% O_2_ hypoxia treatment. ILVs were characterized by Flotillin protein or multi‐transmembrane proteins such as CD63 and CD81.^[^ ^24^ ^]^ Hypoxia significantly increased the number of Flotillin‐positive (Flot^+^) ILVs in primary astrocytes (**Figure** **2**A,B), while having no significant effect on that of primary cerebellar granule neurons (Figure 2C,D) and BV2 microglial cells (Figure 2E,F). Exosomes isolated from the culture medium of both normal and hypoxic‐treated astrocytes using differential ultracentrifugation were further characterized. Their size was determined using qNano (Figure 2G). Transmission electron microscopy (TEM) revealed their typical cup‐shaped morphology and diameters of ≈100 nm (Figure 2H). Western blot results identified astrocytic exosomes by the exosomal markers of CD63, FLOT1, and ALIX (Figure 2I), which were significantly increased by the hypoxia treatment (Figure 2J–L). Moreover, a significant increase in the number of ILVs was observed in astrocytes within the ischemic penumbra area of the cerebral cortex in MCAO mice (Figure 2M,N). Our previous studies showed that crab‐eating macaques exposed to acute hypobaric hypoxia (HH) experienced severe brain damage.^[^ ^25^ ^]^ Consistent with the aforementioned findings, the number of ILVs within astrocytes in the prefrontal cortex of macaques treated with HH was significantly higher compared to the normobaric normoxia (NN) group (Figure S2A,B, Supporting Information). In conclusion, both in vitro and in vivo data demonstrate that hypoxia selectively promotes the biogenesis of exosomes in astrocytes.

### 2.3 HK2 Facilitates the Formation of ILVs in Hypoxia‐Treated Astrocytes Without Affecting Their Lysosomal Degradation

**Figure 3:**
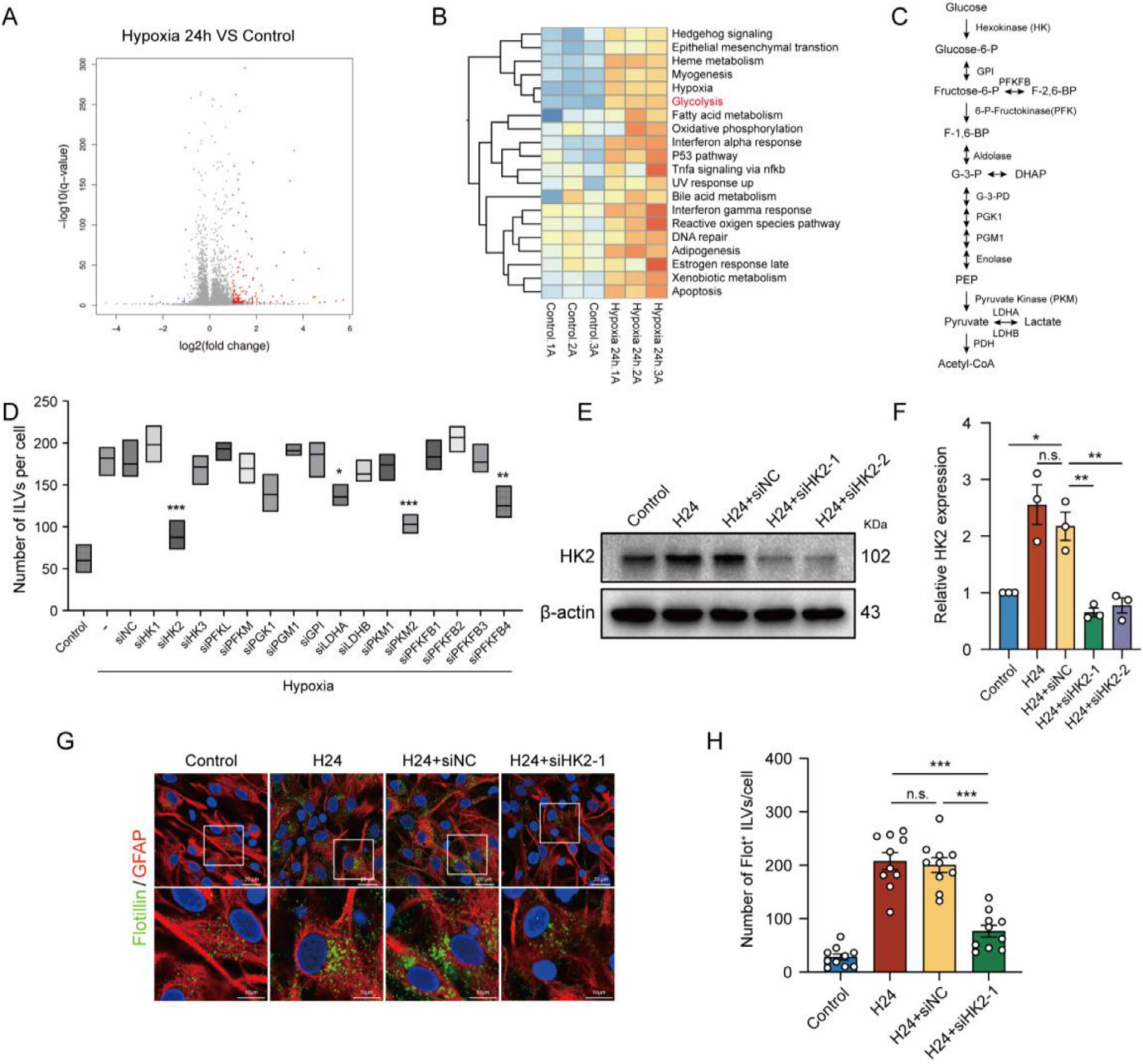
HK2 facilitates the formation of ILVs in hypoxia‐treated astrocytes A) Volcano‐plot of differentially expressed genes (DEGs) between normal and hypoxia‐treated primary astrocytes. B) GO clustering of hallmarks indicates the activation of glycolysis upon hypoxia treatment; C) The glycolysis pathway and gene candidates for the RNAi screening. D) The number of ILVs per astrocyte was quantified following hypoxia treatment, using an RNAi library targeting 16 enzymes in the glycolytic pathway. E) Expression of HK2 in astrocytes infected with HK2 siRNA following hypoxia treatment was analyzed by western blot. F) Quantification of HK2 protein levels normalized to β‐actin and presented as fold‐change relative to the control group in (E). G) Immunofluorescence imaging of ILVs (Flot^+^, green) in primary astrocytes (GFAP, red) treated with HK2 siRNA following hypoxia treatment, bar = 20 µm (up), bar = 10 µm (down). H) Statistical analysis number of ILVs per cell in (G). Data were presented with Mean ± SEM. Statistical analyses were performed using one‐way ANOVA followed by Tukey's multiple comparison tests; n.s., no significance. ^*^ *p* < 0.05; ^**^ *p* < 0.01, ^***^ *p* < 0.001.

To further investigate the key pathways and regulators involved in hypoxia‐induced exosomes release, RNA‐Seq transcriptomic analysis was performed on astrocytes cultured under normal conditions and 1% O_2_ hypoxia for 24 h. The results revealed significant upregulation of numerous genes following hypoxia (**Figure** **3**A). GO functional clustering demonstrated a marked enhancement of the glycolytic pathway in hypoxia‐treated astrocytes (Figure 3B). To identify specific glycolytic enzymes involved in regulating astrocytic exosomes production, an RNA interference library targeting 16 glycolytic enzymes was employed (Figure 3C). Immunofluorescence results showed that the knockdown of HK2, lactate dehydrogenase A (LDHA), PKM2, and 6‐phosphofructo‐2‐kinase/fructose‐2,6‐biphosphatase 4 (PFKFB4) all reduced the number of ILVs, with the most significant effect observed for HK2 (Figure 3D). We have previously demonstrated that microglial HK2 mediates inflammatory brain injury in acute ischemic stroke.^[^ ^26^, ^27^ ^]^ Here, we confirmed that hypoxia stimulated the remarkable upregulation of HK2 in astrocytes (Figure 3E,F). Furthermore, the knockdown of HK2 by siRNA significantly decreased the number of ILVs in astrocytes (Figure 3G,H). In summary, these results demonstrate that HK2 is a key regulator involved in promoting hypoxia‐induced ILVs production and exosomes release in astrocytes.

**Figure 4:**
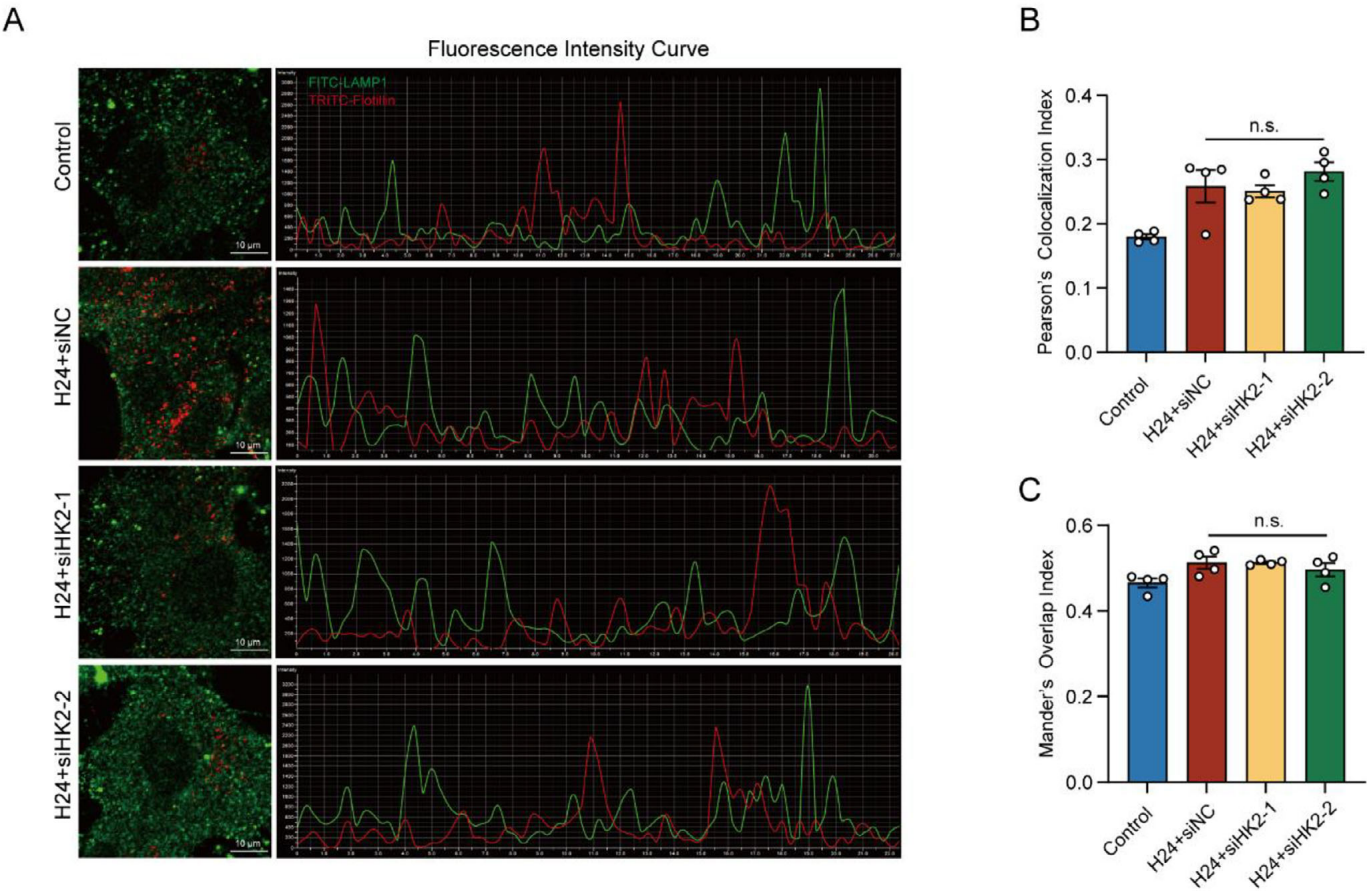
HK2 does not affect the co‐localization of ILVs with lysosomes following hypoxia treatment. A) Immunofluorescence double‐labeled staining of ILVs marker flotillin‐1 (red) and lysosomes marker LAMP1 (green) in primary rat astrocytes infected with HK2 siRNA or siNC following hypoxia treatment. The fluorescence intensity curves were shown to detect the colocalization of lysosomes and ILVs. B,C) The statistical analysis of PCI B) and MOI C). Data were presented with Mean ± SEM. Statistical analyses were performed using one‐way ANOVA followed by Tukey's multiple comparison tests. n.s., no significance.

To further investigate whether HK2 is involved in the degradation process of ILVs, we analyzed the co‐localization of Flotillin and lysosome marker LAMP1 under normoxic and hypoxic conditions with HK2 interference. Immunofluorescence analysis revealed no significant co‐localization between Flotillin‐labeled ILVs and LAMP1 in either condition (**Figure** **4**A). Furthermore, the fluorescence intensity curves of both signals in each group did not exhibit synchronous trends, confirming the lack of co‐localization between ILVs and lysosomes (Figure 4A). In addition, no significant difference was observed in the Mander's Overlap Index (MOI) (Figure 4B) or the Pearson's Colocalization Index (PCI) (Figure 4C) among the groups. These results suggest that HK2 does not affect the degradation process of ILVs within lysosomes.

### 2.4 HK2 Directly Binds to and Phosphorylates nSMase1

**Figure 5:**
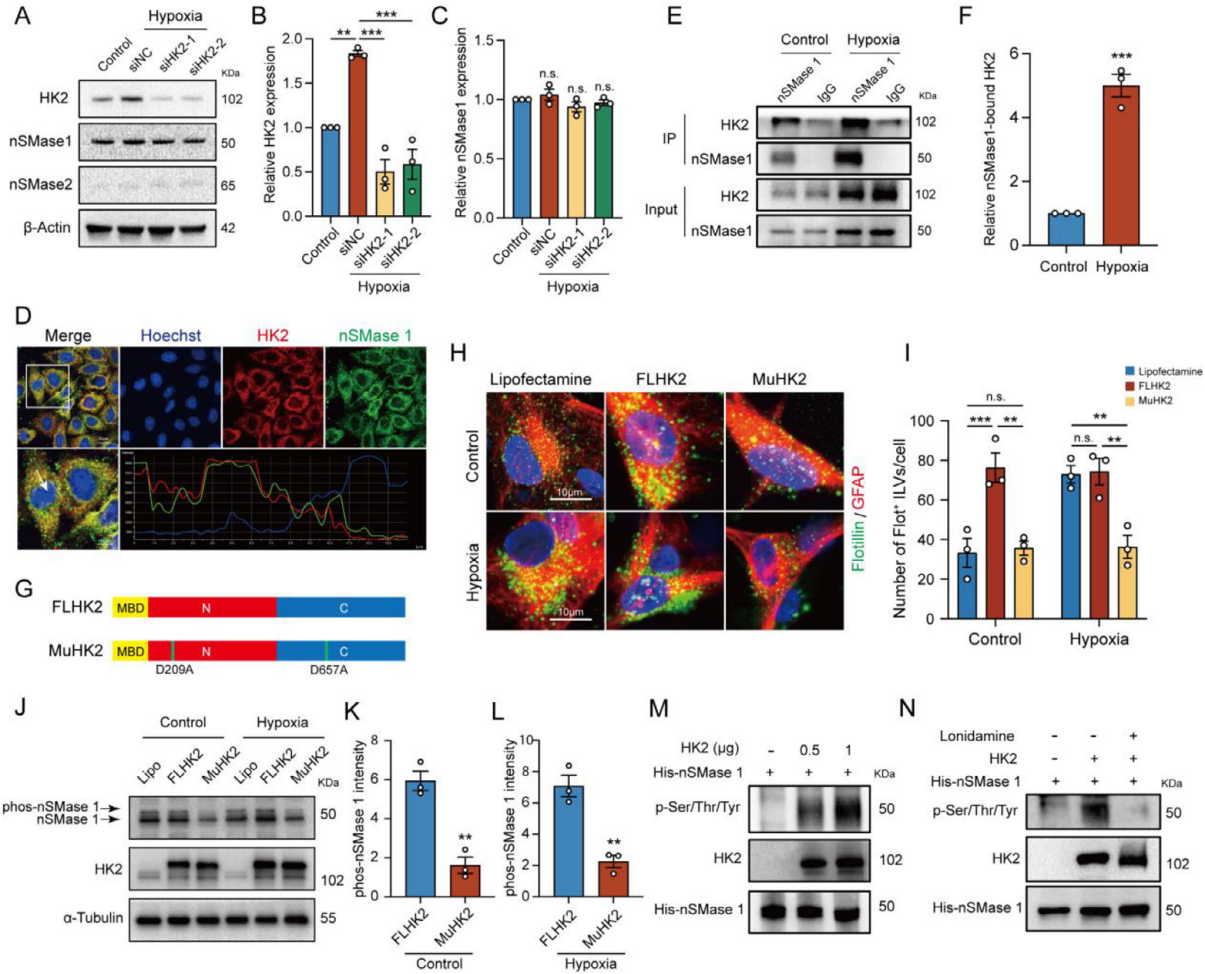
HK2 directly binds to and phosphorylates nSMase1. A) Protein expression of HK2, nSMase1 and nSMase2 in astrocytes transfected with siHK2‐1 and siHK2‐2. B,C) Quantification of HK2 B) and nSMase1 C) protein levels normalized to β‐actin and presented as fold‐change relative to the control group in (A). D) The immunofluorescence imaging of nSMase1 and HK2 in human astrocyte cell line. The fluorescence intensity curve of HK2 (TRITC) and nSMase1 (FITC) was shown to indicate the colocalization of HK2 and nSMase1 in primary human astrocytes, bar = 20 µm (up), bar = 10 µm (down). E) CO‐IP confirmed that nSMase1 interacted with HK2 under hypoxia. F) Relative nSMase1‐bound HK2 normalized to the control group. G) The structural scheme of FLHK2 and MuHK2. H) Representative immunofluorescence images of Flotillin‐1‐labeled ILVs (green) in primary human astrocyte (GFAP, red) transfected with empty vector, FLHK2 or MuHK2 vectors, bar = 10 µm. I) Statistical analysis of ILVs numbers of astrocytes in (H). J) Phos‐tag western blot of cell lysates of human astrocytes transfected with empty vector, FLHK2 or MuHK2 vectors. K,L) Quantification of phos‐nSMase1 band intensity (normalized to FL‐HK2 group) was performed under control K) and hypoxia L) conditions. (M and N) HEK293T cells transfected with pcDNA3.1‐His‐nSMase1 were lysed and subjected to immunoprecipitation using anti‐His magnetic beads, and then purified His‐nSMase1 protein was incubated with varying amounts of recombinant HK2 protein in vitro M) or with/without lonidamine N) at 37 °C for 1 h. The reaction mixtures were then subjected to Western blotting and probed with anti‐Phosphoserine/threonine/tyrosine antibody. Data were presented with Mean ± SEM. Statistical analysis in (F), (K) and (L) was performed by unpaired Student's t test. Statistical analysis in (B), (C) and (I) was performed using one‐way ANOVA followed by Tukey's multiple comparison tests. n.s., no significance. ^*^ *p* < 0.05; ^**^ *p* < 0.01; ^***^ *p* < 0.001.

The ceramide‐mediated lipid pathway is a stress response activated by cellular challenges such as inflammation and oxidative stress. nSMase catalyzes the hydrolysis of sphingophospholipids to form intracellular ceramides and is robustly activated under various environmental stress conditions.^[^ ^28^, ^29^ ^]^ It has been found that inhibiting nSMase1 and nSMase2 activity can significantly reduce exosomes production in various cell types.^[^ ^8^, ^30^, ^31^ ^]^ To investigate the potential regulatory effects of HK2 on nSMase, we first assessed the protein levels of nSMase1 and nSMase2 in astrocytes. Western blot results showed that the expression of nSMase2 was hardly detectable and significantly lower than that of nSMase1 (**Figure** **5**A), suggesting that nSMase1 is responsible for the hypoxia‐induced exosomes production in astrocytes. However, knockdown of HK2 using siRNA did not affect the protein level of nSMase1 (Figure 5B,C). Immunofluorescence analysis revealed significant overlap of the HK2 and nSMase1 signals in human astrocytes, with synchronized fluorescence intensity changes, indicating a spatial co‐localization between HK2 and nSMase1 (Figure 5D). We confirmed the interaction between HK2 and nSMase1 by co‐immunoprecipitation (CO‐IP), which was significantly enhanced by hypoxia treatment (Figure 5E,F). Given the phosphorylated nSMase1 represents its activity form,^[^ ^32^ ^]^ this interaction suggests the possibility for HK2 to phosphorylate nSMase1. We constructed the eukaryotic expression plasmid of MuHK2, containing D209A/D657A mutations with inactivate kinase function,^[^ ^33^ ^]^ and that of the full‐length HK2 (FLHK2) (Figure 5G) to investigate the impact of HK2 kinase activity on ILVs formation. Overexpression of FLHK2 significantly promoted ILVs formation even under normoxic conditions, whereas transfection with the MuHK2 mutant markedly decreased the numbers of Flotillin‐1 positive ILVs both under normoxic and hypoxic conditions (Figure 5H,I). These results suggest that hypoxia‐induced ILVs formation in astrocytes is dependent on the kinase activity of HK2. We further detected the phosphorylation of nSMase1 by HK2 using phos‐tag gel electrophoresis. The phosphorylation levels of nSMase1 were significantly reduced in astrocytes transfected with the MuHK2 plasmid, as compared to those transfected with the FLHK2 plasmid, regardless of normoxic (Control) or hypoxic conditions (Figure 5J–L). An in vitro kinase assay showed that recombinant human HK2 protein directly phosphorylated nSMase1 (Figure 5M). Notably, this phosphorylation was abolished by the HK2 inhibitor lonidamine (Figure 5N). Together, these results demonstrate that HK2 mediates hypoxia‐induced exosomes production in astrocytes by phosphorylating nSMase1 directly.

### 2.5 Exosomes Released by Hypoxia‐Induced Astrocytes Disrupt Cerebrovascular Endothelial Tight Junctions

**Figure 6:**
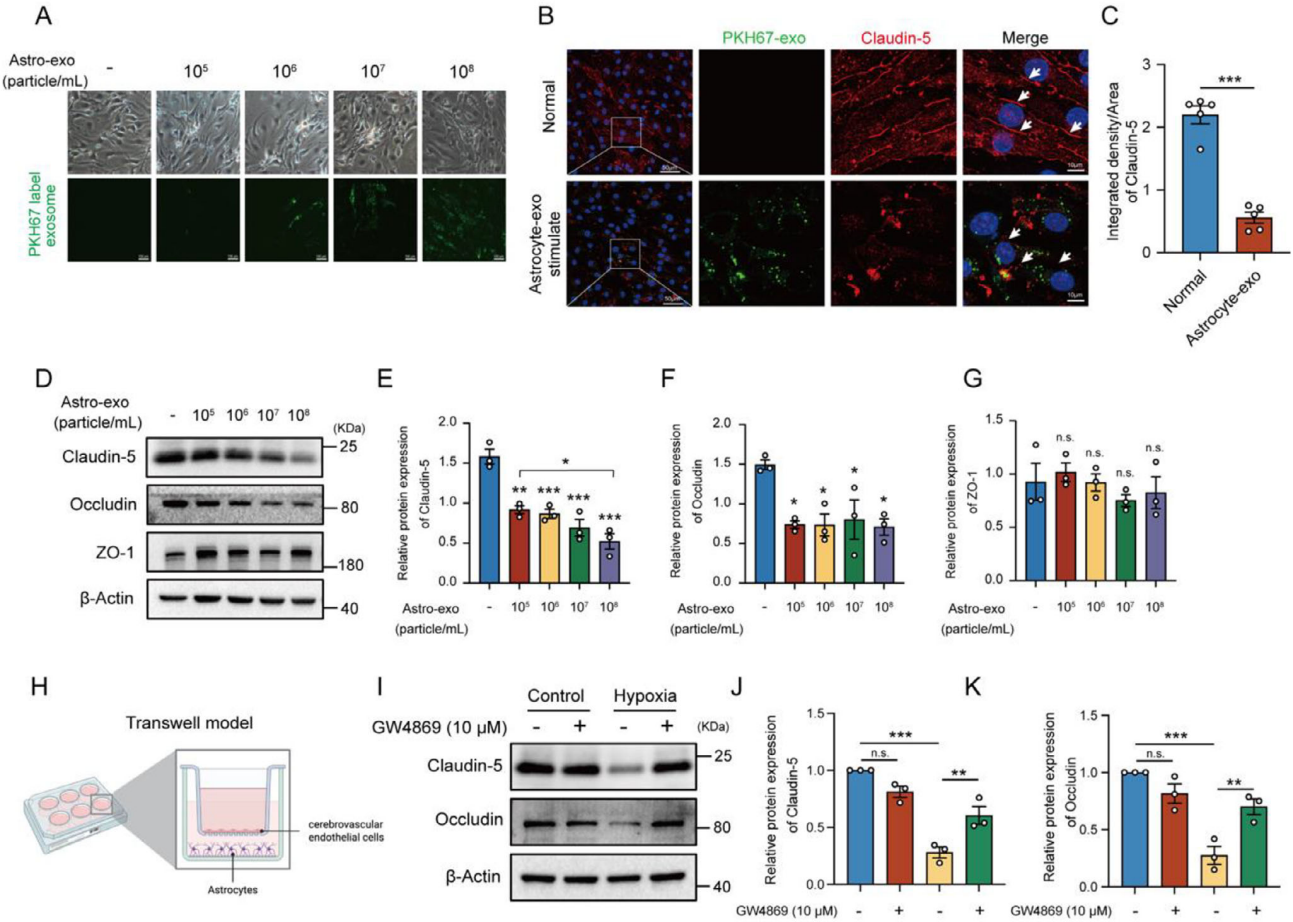
Exosomes released by hypoxia‐induced astrocytes disrupt cerebrovascular endothelial tight junctions. A) Exosomes released by hypoxia‐induced astrocytes were isolated and labeled with PKH‐67 dye, then co‐incubated with cerebrovascular endothelial cells bEND.3 for 24 h. Fluorescence microscopy was used for observation, bar = 100 µm. B) After 24 h of co‐incubation with hypoxia‐induced astrocyte‐derived exosomes, representative confocal immunofluorescence images of PKH‐67 (green) and claudin‐5 (red) double‐staining in bEND.3 cells are shown, with or without astro‐exo treatment. White arrows indicate the status of tight junctions, bar = 50 µm (left), bar = 10 µm (right). C) Quantification of the average fluorescence intensity of claudin5 in (B). D) Following 24 h of treatment with different concentrations of astro‐exo, the expression of tight junction proteins ZO‐1, Occludin, and Claudin‐5 was detected by Western blot. E–G) Protein expression levels of claudin‐5 E), Occludin F), and ZO‐1 G) were normalized to β‐actin and statistically analyzed as shown in (D). H) Schematic diagram of the transwell model. Created in https://BioRender.com. I) The astrocytes‐cerebrovascular endothelial cells co‐culture system was treated with or without GW4869, followed by 24‐h hypoxia exposure. The protein expression of Occludin and Claudin‐5 was analyzed by Western blot. The protein expression levels of Claudin‐5 J) and Occludin K) were normalized to β‐actin and statistically analyzed as shown in (I). Data were presented as Mean ± SEM. Statistical analysis in (C) was performed by unpaired Student's *t*‐test. Statistical analysis was performed using one‐way ANOVA followed by Tukey's multiple comparison tests. n.s., no significance. ^*^ *p* < 0.05; ^**^ *p* < 0.01. ^***^ *p* < 0.001.

Astrocytes are a key component of the blood‐brain barrier (BBB), with their astrocytic end feet structurally attached to the endothelial cells of blood vessels, forming the neurovascular unit together with endothelial cells and pericytes. In response to brain injury, reactive astrocytes promote BBB permeability by releasing various cytokines, chemokines, and other factors.^[^ ^34^ ^]^ To investigate the potential effects of astrocyte‐derived exosomes on cerebrovascular endothelial cells, hypoxia‐induced exosomes from primary astrocytes were isolated and then labeled with PKH‐67 dye. After incubating bEND.3 cerebrovascular endothelial cells with varying concentrations of astrocyte‐derived exosomes (astro‐exo) induced by hypoxia for 24 h, significant internalization of astro‐exo in endothelial cells was observed (**Figure** **6**A). Claudin‐5 is a crucial tight junction protein in endothelial cells, particularly within the BBB. Immunofluorescence analysis revealed notable disruption of Claudin‐5 in endothelial cells that internalized astro‐exo, in contrast to the continuous expression observed in the control group (Figure 6B,C). Occludin is a transmembrane protein critical for tight junction formation, while Zonula Occludens‐1 (ZO‐1) is a cytoplasmic scaffolding protein vital for their assembly and maintenance. Western blot analysis of tight junction proteins in endothelial cells incubated with astro‐exo showed a significant downregulation of Claudin‐5 and Occludin, while ZO‐1 levels remained unaffected (Figure 6D–G). A transwell co‐culture system was further employed with astrocytes residing in the lower chamber and cerebrovascular endothelial cells in the upper compartment (Figure 6H). Western blot analysis demonstrated that hypoxia significantly downregulated both tight junction protein levels of Occludin and Claudin‐5 in endothelial cells, while administration of GW4869, an inhibitor of exosome synthesis and release, substantially restored their expression levels under hypoxic conditions (Figure 6I–K). These results suggest that exosomes released by hypoxia‐induced astrocytes contribute to the disruption of endothelial tight junctions.

### 2.6 Selective Knockdown of Astrocytic HK2 Reduces Exosomes Release and Alleviates MCAO‐Induced Brain Injury

**Figure 7:**
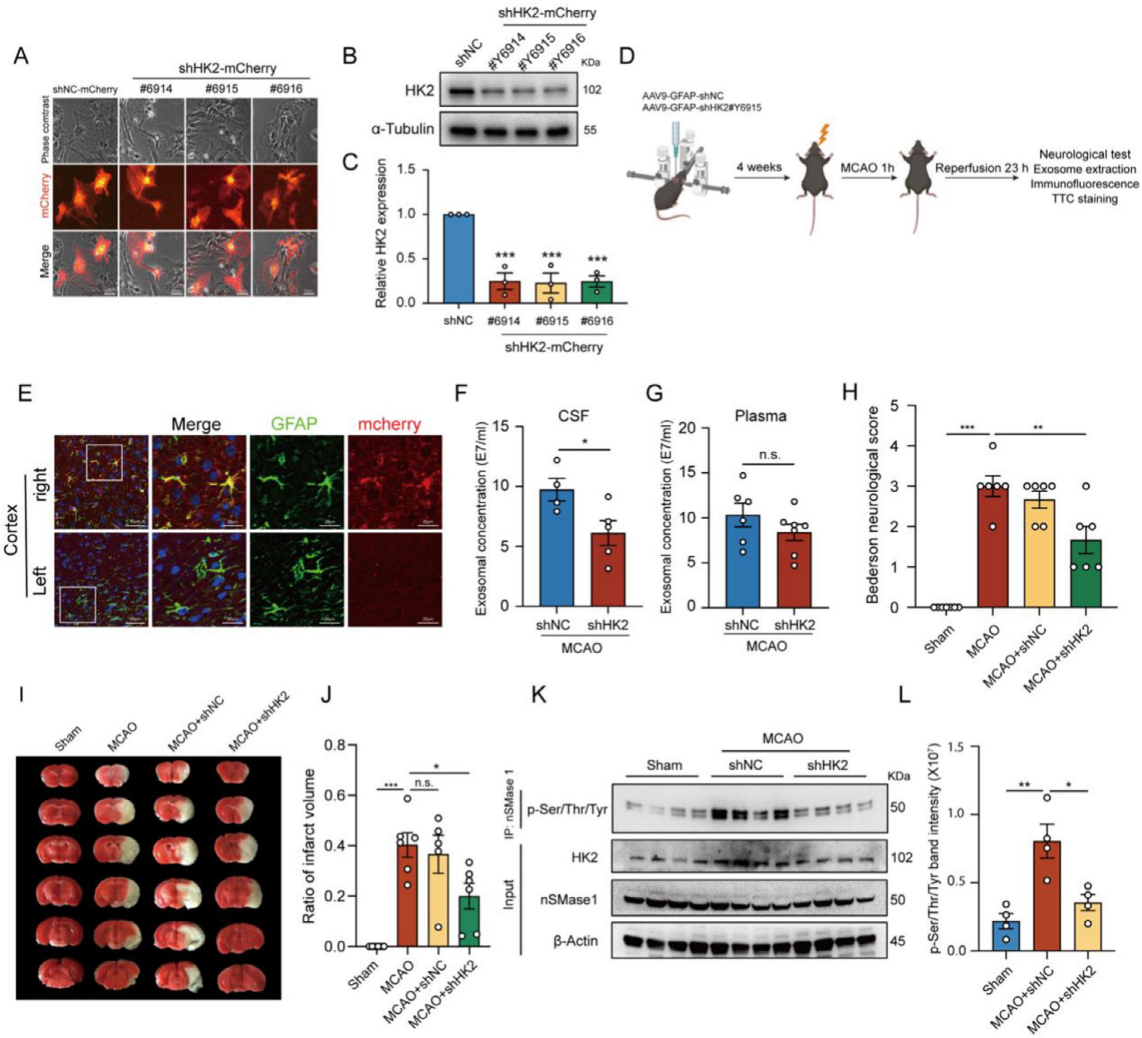
Selective knockdown of astrocytic HK2 in mice reduces exosomes release and mitigates MCAO‐induced brain injury. A) The infection of 10^6^ v.g./mL AAV9 viruses for 7 days on primary mouse astrocytes in vitro, bar = 100 µm. B) Western blot analysis of HK2 in primary mouse astrocytes infected with AAV9‐shHK2. C) Quantification of HK2 protein levels normalized to α‐Tubulin and presented as fold‐change relative to the shNC group in (B). D) Experimental schematic diagram. Created in http://BioRender.com. E) The immunofluorescence imaging of mCherry‐labeled AAV9 viruses (red) and GFAP‐labeled astrocytes (green) in the infected cortex of the mouse brain, bar = 50 µm (left), bar = 20 µm (right). F,G) The statistical analysis of exosomal concentrations in CSF (E) and plasma F) respectively. H) The Berdeson's neurological scores of mice in different groups. I) TTC staining of brain slices in mice of different groups indicating the infraction of brain tissues. J) The ratio of infarct volume of mouse brains of different groups. K) Homogenates from the ipsilateral hemisphere of MCAO‐operated mice were subjected to co‐immunoprecipitation using anti‐nSMase1 antibody conjugated to protein A/G magnetic beads. Phosphorylation levels of nSMase1 were assessed by western blot with anti‐Phosphoserine/threonine/tyrosine antibody. L) Quantification of phos‐nSMase1 band intensity was performed. Data were presented as Mean ± SEM. Statistical analysis in (F) and (G) was performed by an unpaired Student's *t*‐test. Statistical analysis in (C), (H), (J) and (L) was performed using one‐way ANOVA followed by Dunnett's post hoc analysis. n.s., no significance. ^*^ *p* < 0.05; ^**^ *p* < 0.01. ^***^ *p* < 0.001.

To investigate the potential role of HK2‐induced exosomes released from astrocytes in acute ischemic stroke, an adeno‐associated virus (AAV) vector specifically targeting HK2 in mouse astrocytes was constructed (pAAV9‐shortGFAP‐miR30 shHK2‐mCherry). Following a 7‐day infection with a titer of 10^6^ v.g./mL, all three kinds of shHK2 constructions significantly infected primary astrocytes (**Figure** **7**A) and effectively downregulated HK2 expression (Figure 7B,C). AAV9‐shHK2 (Y6915) was selected to stereotactically inject into the right subcortical region of C57BL/6 mice, while the left subcortical region, injected with saline, served as the control. After the injections, a 4‐week period was allowed before performing MCAO surgery (Figure 7D). Immunofluorescence analysis confirmed that AAV9‐shHK2 selectively infected astrocytes in vivo (Figure 7E). Knockdown of HK2 significantly reduced the number of exosomes in the CSF of MCAO mice 24 h postsurgery (Figure 7F), with a decreasing trend observed in plasma‐derived exosomes (Figure 7G). Bederson's neurological function score was used to assess motor function and limb coordination in the mice. MCAO induced significant motor dysfunction, whereas MCAO mice infected with AAV9‐shHK2 exhibited significantly lower Bederson scores (Figure 7H). TTC staining revealed significant cerebral infarction in the right hemisphere of MCAO mice, which was markedly diminished in those infected with AAV9‐shHK2 (Figure 7I,J). Furthermore, co‐immunoprecipitation analysis of ischemic hemisphere homogenates demonstrated that MCAO significantly enhanced nSMase1 phosphorylation, which was profoundly abrogated by astrocyte‐specific HK2 knockdown (Figure 7K,L). Collectively, these findings indicate that specific inhibition of astrocytic HK2 significantly reduced exosomes release and alleviated brain injury in MCAO mice.

## 3 Discussion

Enhanced glycolysis has been shown to be associated with a poorer prognosis in acute ischemic stroke,^[^ ^35^, ^36^ ^]^ with its pathological effects attributed to metabolic byproducts including lactate‐induced intracellular acidosis lactate,^[^ ^37^ ^]^ protein lactylation^[^ ^38^ ^]^ and NADPH^[^ ^39^, ^40^ ^]^ mediated oxidative stress. Furthermore, glycolytic enzymes PKM2^[^ ^41^ ^]^ and 6‐phosphofructo‐2‐kinase/fructose‐2,6‐biphosphatase 3 (PFKFB3)^[^ ^42^ ^]^ have been shown to contribute to ischemic brain damage via neuroinflammation and energy metabolism. We previously reported that inhibition of HK2 in microglia reduces the inflammatory brain injury during acute ischemic stroke.^[^ ^26^, ^27^ ^]^ In this study, selective HK2 inhibition in astrocytes reduced the infarct volume and improved functional outcomes after MCAO surgery. HK2 is an inducible isoform of the hexokinase family under disease conditions including ischemic stroke. Selectively inhibition of HK2 is expected to alleviate brain injury without interfere with the normal brain energy metabolism. Moreover, selectively suppression of the initial glycolytic step catalyzed by HK2 may also attenuate the increased metabolic flux of the pentose phosphate pathway, which mediates the oxidative stress injury induced by NADPH oxidase.^[^ ^39^, ^40^ ^]^ Collectively, this study, in conjunction with our previous reports, establish HK2 as a pivotal injury molecule in ischemic stroke.

The exosomes derived from astrocytes may serve as a double‐edged sword in ischemic stroke. Several studies indicate that astrocytic exosomes exert neuroprotective effects in the recovery phase of stroke. For instance, inhibition of extracellular vesicles with CD38 siRNA on post‐surgical day 5 significantly increases infarct volume and exacerbates neuronal damage.^[^ ^43^ ^]^ However, we report here that hypoxia‐induced astrocytic exosomes may contribute to BBB disruption during acute ischemic injury. In align with our observation, inflammatory cytokine IL‐1β stimulates astrocytes to release exosomes, which promote the infiltration of inflammatory cells, such as neutrophils, thereby inducing inflammatory damage.^[^ ^44^ ^]^ Furthermore, stimulation of astrocytes by TNF‐α and IL‐1β leads to the release of exosomes containing Exo‐miR‐125a‐5p, which inhibit dendritic growth and neuronal electrical activity.^[^ ^45^ ^]^ Since the functional properties of exosomes are intimately associated with their cargo composition, a systematic analysis of the composition of astrocyte‐derived exosomes holds significant importance for elucidating their multifaceted roles in distinct pathophysiological processes during cerebral stroke. Additionally, post‐activation reactive astrocytes polarize into dual phenotypes (A1/A2), exerting divergent effects on BBB integrity. In the acute phase of cerebral ischemia, A1 astrocytes secrete cytokines such as vascular endothelial growth factor (VEGF) that damages endothelial cells and increases BBB permeability. Conversely, A2 astrocytes protect BBB integrity through neuroprotective mediators such as pentraxin 3 (PTX3) and insulin‐like growth factor‐1 (IGF‐1).^[^ ^46^ ^]^ Our data reveal that acute‐phase exosomes in ischemic stroke primarily derive from astrocytes, suggesting a potential regulatory role of HK2 in A1 astrocyte polarization.

HK2 catalyzes the initial step of glycolysis by phosphorylating glucose to glucose‐6‐phosphate. Beyond its canonical role in energy metabolism,^[^ ^47^, ^48^ ^]^ HK2 has been implicated in regulating the mitochondrial permeability and apoptosis via interaction with voltage dependent anion channel 1 (VDAC1),^[^ ^49^ ^]^ modulating autophagy through binding to mammalian target of rapamycin (mTORC1),^[^ ^50^ ^]^ and maintaining cell stemness through subcellular localization dynamics.^[^ ^51^ ^]^ Notably, emerging evidence from yeast studies suggests HK2 may possess protein kinase activity,^[^ ^52^, ^53^ ^]^ a function recently linked to immune escape of tumors where mitochondrial dissociation of HK2 activates NF‐κB signaling via phosphorylation of IκB‐α.^[^ ^54^ ^]^ Here we demonstrate that HK2 interacts with nSMase1 and directly phosphorylate it to agitate exosome biogenesis. Given the widespread involvement of HK2 and its family members in diverse physio‐pathological processes, identification of HK2's protein kinase activity toward nSMase1 may provide novel mechanistic insights into the pathogenesis of these diseases.

nSMase is broadly recognized as the key enzyme in the lipid pathway, catalyzing the ceramide generation to facilitate lipid raft formation and membrane invagination during ILV biosynthesis. Both nSMase1 and nSMase2 contribute to the biogenesis of exosome.^[^ ^8^, ^30^, ^32^ ^]^ However, our data show undetectable nSMase2 protein levels in astrocytes. We provide preliminary evidence that HK2 promotes hypoxia‐induced astrocytic exosome production by activating nSMase1. Whether HK2 regulates nSMase2 to participate in exosome production in other cell types requires further investigation. In addition to lipid‐mediated mechanisms, exosome biogenesis is also associated with the ESCRT‐I and ESCRT‐II complexes, which drive endosomal membrane inward budding.^[^ ^19^, ^55^ ^]^ Intriguingly, in budding yeast, ESCRT complex plays a crucial role in lipophagy during glucose starvation,^[^ ^56^ ^]^ implying a potential crosstalk between hexokinase and ESCRT machinery in exosome biogenesis. Additionally, exosome production involves multiple sequential steps, including the formation of early endosomes, maturation into late endosomes/MVBs, transport of MVBs to the plasma membrane, and the release of exosomes following fusion with the plasma membrane.^[^ ^1^ ^]^ Our results showed that HK2 did not affect lysosomal degradation of ILVs or MVBs, but whether it regulates the other steps remains to be further investigated.

## 4 Experimental Section

### Human Samples

The stroke blood samples were obtained from stroke patients at the Third Affiliated Hospital of Sun Yat‐sen University, while normal blood samples were collected from healthy volunteers. This study was approved by the Medical Ethics Committee of the Third Affiliated Hospital of Sun Yat‐sen University (Approval No. (2022) 02‐015‐01).

### Animals

All animal experiments were performed according to the regulations approved by the Animal Care and Ethics Committee of Sun Yat‐sen University. All animals were housed in a light/dark cycle with food and water available. Animal experiments were conducted following the Guidelines for the Care and Use of Laboratory Animals of Sun Yat‐Sen University (the protocol number is SYSU‐IACUC‐2020‐B0068). Newborn C57BL/6J mice were purchased from Laboratory Animals of Sun Yat‐Sen University. Eight‐week‐old male C57BL/6J Mice were purchased from GemPharmatech Co., Ltd (Jiangsu, China).

Six‐year‐old male cynomolgus monkeys (6.8–7.5 kg) were obtained from Gaoyao Kangda Laboratory Animals Science & Technology Co., Ltd, Zhaoqing, Guangdong Province, China. The animals were transported to the Third Military Medical University, Chongqing, China. Monkeys were singly housed in cages in a controlled environment at a temperature of 25 ± 1 °C, relative humidity of 60%, and circadian 12 h light/dark cycle and were fed routinely. The experimental protocols were approved by the Ethics Committee of Zhongshan School of Medicine, Sun Yat‐Sen University (Number 57 2014)

### Exo‐Free FBS Preparation

To eliminate the interference of exosomes from fetal bovine serum (FBS), the FBS was processed through ultracentrifugation to pellet the exosomes, resulting in exosome‐free serum (Exo‐free FBS) for cell culture. The preparation of Exo‐free FBS is as follows, 38.5 mL of FBS was poured into a sterile heat‐sealed tube and centrifuged overnight at 100 000 g. After centrifugation, the supernatant was aseptically collected as Exo‐free FBS.

### Cell Culture

BV2 microglia cell line is purchased from the China Center for Type Culture Collection (CCTCC). Human astrocyte cell line is purchased from ScienCell Research Laboratory. 293T cell line is purchased from the American Type Culture Collection (ATCC). The cell lines were cultured in the appropriate medium containing 10% Exo‐free FBS, following the manufacturer's instructions. The cells were incubated in a humidified incubator at 37 °C with 5% CO_2_.

### Primary Cell Isolation and Culture

For primary astrocyte, cortex was dissected from a mouse at postnatal days 1–2. Following tissue mincing, it was subjected to digestion in trypsin for 15 min, after which the process was halted by the addition of a digestion solution containing 0.1% DNase I (Sigma Aldrich, D5025). The mixture was then centrifuged at 1000 rpm for 5 min to remove the supernatant. Subsequently, an appropriate volume of 0.1% DNase I digestion solution was added to obtain a single‐cell suspension. Cells were resuspended and cultured in DMEM (Gibco,11965‐118) containing 10% FBS at 37 °C. After 24 h, the medium was replaced. The medium was replaced every 2–3 days. After 7 days of culture, mixed glial cells were cultured at 37 °C and shaken overnight at 230 r min⁻¹ to remove the suspended microglia, resulting in a higher purity of astrocytes for subsequent experiments.

For cerebellar granule neurons, Cerebellums were dissected from mouse at postnatal days 7–8, trypsinized, and dissociated in trypsin and 0.1% DNase I. Collected the upper layer of the single‐cell suspension and centrifuged at 1000 rpm for 5 min to collect the cell pellet. Cells were resuspended and cultured in BME (Gibco, 21010046) containing 10% FBS at 37 °C. After 24 h of culture, adding 10 µM Ara‐C (Sigma Aldrich, C1768) to suppress the proliferation of glial cells. Supplement with 5 mm glucose (Gibco, A2494001) on day 7. After 8 days of culture, mature neurons were collected.

### Hypoxia Treatment

Cells are placed in a HypoxyLab hypoxia workstation for hypoxic treatment (37 °C, controlled relative humidity at 90%, oxygen concentration at 1%, and carbon dioxide concentration at 5%). Subsequent procedures are performed after the cells are treated for the designated duration.

### Middle Cerebral Artery Occlusion (MCAO) Model

Before the operation, 10‐week‐old male C57BL/6 mice were fasted overnight but were given drinking water. The next day, the mice were weighed and anesthetized with isoflurane. During the surgery, body temperature was maintained at 37 ± 0.5 °C using a heating pad. The unilateral common carotid artery and external carotid artery were then exposed and ligated. A 0.28‐mm nylon monofilament with a silicone‐coated tip was inserted into the internal carotid artery, thereby occluding the origins of the anterior cerebral and middle cerebral arteries. After 60 min of ischemia, reperfusion was accomplished by withdrawing the nylon monofilament. All surgeries were performed under anesthesia. After the surgery, the animals were given agar gel. After 23 h of reperfusion, the neurobehavioral scores of the mice were determined according to the Berdeson scoring. Then, the mice were anesthetized with isoflurane and perfused with normal saline and paraformaldehyde, and the brain tissue was collected. All efforts were made to minimize animal suffering and to reduce the number of animals used.

### CSF Collection

In brief, mice were anesthetized with isoflurane gas and then placed in a stereotactic instrument. The skin and muscle were separated to expose the dura mater without any bleeding, through which the cisterna magna was visible. A glass microelectrode tube with a diameter of 0.5 mm was used to puncture the dura mater and aspirate the CSF, which appeared clear and slightly yellowish. After collection, 1 mL of saline was injected subcutaneously to prevent dehydration, and the muscle and skin were sutured.

### Exosomes Preparation and Isolation

For ultracentrifugation, the culture medium was centrifuged at 300 × *g* for 10 min and then at 2000 × g for 10 min to remove the cells and other debris, followed by centrifugation at 10 000 × g for 30 min to remove large vesicles. Then, the supernatant was centrifuged at 100 000 × g for 70 min. Exosomes were collected from the pellet and resuspended in FBS‐free medium for subsequent experiments.

### Density Gradient Ultracentrifugation

Blood samples were collected using EDTA as an anticoagulant. The samples were centrifuged at 2500 rpm for 10 min at room temperature to obtain plasma, followed by centrifugation at 5000 g for 10 min at room temperature to remove fibrin precipitates, and the supernatant was collected. The supernatant was then centrifuged at 10 000 g for 30 min at 4 °C to remove contaminating proteins and impurities, and the supernatant was transferred to a new ultracentrifuge tube. Next, ultracentrifugation was performed at 120 000 g for 90 min at 4 °C, and the exosomes pellet was resuspended in a PBS solution. A four‐layer density gradient iodixanol solution was prepared with densities of 1.054, 1.079, 1.127, and 1.223 g mL^−1^ and subjected to ultracentrifugation at 150 000 g for 12 h at 4 °C overnight. The centrifuged density gradient solution was fractionated into six gradients within the range of 1.13–1.19 g mL^−1^, sequentially numbered from 1 to 6 from low to high density, and then transferred to new ultracentrifuge tubes. PBS was used to fill the ultracentrifuge tubes for each fraction, followed by ultracentrifugation again at 120 000 g for 90 min at 4 °C. The supernatant was carefully discarded to obtain exosomes from each density layer.

### ExoQuick Method

Exosomes from mouse cerebrospinal fluid were extracted following the instructions provided with the ExoQuick Exosomes Precipitation Kit (System Biosciences, EXOQ5TM‐1). ExoQuick reagent was added to the cerebrospinal fluid supernatant at a 5:1 ratio and allowed to stand at 4 °C for 30 min. The mixture was then centrifuged at 1500 g for 30 min at 4 °C to collect the pellet. The pellet was resuspended in PBS and passed through a 0.22 µm filter for collection.

### qNano

In this study, the qNano nano‐particle analysis system was used to characterize the particle count and size distribution of exosomes. The exosomes samples obtained were resuspended in PBS and placed on ice. The qNano instrument (Izon Sciences Ltd.) was then activated to measure the particle size of the exosomes. Testing was then performed, starting with the CPC‐100 standard, followed by the samples. Subsequently, the data were analyzed using Izon Control Suite software, and corresponding images were generated.

### PKH67 Staining

Exosomes were stained with the PKH67 Green Fluorescent Cell Linker Kit (Sigma‐Aldrich), according to the manufacturer's instructions with minor modifications.

### Transmission Electron Microscopy (TEM) Assay

Transmission electron microscopy for exosomes characterization was performed by OBiO Technology Co., Ltd. (Shanghai).

### Western Blot

Total cellular proteins were extracted using M‐PER lysis buffer (Thermo Fisher Scientific, 78503). Protein concentrations were determined by the BCA assay (Thermo Fisher Scientific, 23225). The extracts were subjected to SDS–PAGE. The membranes were blocked with 5% BSA, followed by incubation with primary antibodies and species‐appropriate horseradish peroxidase‐conjugated secondary antibodies. The primary antibodies used including anti‐CD63 (abcam, ab217345), anti‐CD81 (CST, 10073), anti‐ALIX (Novus, NBP1‐90201), anti‐Calnexin (abcam, ab22595), anti‐α‐Tubulin (CST, 2125), anti‐Flotillin (CST, 18634), anti‐nSMase (abcam, ab131330), anti‐β‐Actin (CST, 4970), anti‐HK2 (abcam, ab209847), anti‐ZO‐1 (abcam, ab96587), anti‐Occludin (Invitrogen, 33–1500), anti‐Claudin 5 (Invitrogen, 35–2500), anti‐Phosphoserine/threonine/tyrosine (Invitrogen, 61–8300). The secondary antibodies used include goat antimouse IgG (HRP) (Arigo, arg65350) and goat anti‐rabbit IgG (HRP) (Arigo, arg65351). Chemiluminescence imaging was performed, and densitometric scanning was conducted using ImageLab software.

### In Vitro Kinase Assay

The pcDNA3.1‐His‐nSMase1 plasmid (encoding human his‐tagged nSMase1) was transfected into 293T cells for 48 h followed by lysed using IP lysis buffer (Beyotime, P0013). His‐nSMase1 protein was harvested by immunoprecipitation with Anti‐his magnetic beads (Beyotime, P2135) at 4 °C with gentle rotation overnight. Beads were subsequently washed three times with cold lysis buffer and then incubated with increasing amounts of recombinant human HK2 protein (Novus, 8179‐HK‐020) with or without lonidamine (100 µm) in 80 µL kinase reaction buffer (1 X Kinase Buffer (CST, 9802), 200 µm ATP (CST, 9804) in the present of protease/phosphatase inhibitor cocktail (TargetMol, C0001) at 37 °C for 1 h. Reactions were terminated by SDS loading buffer and then subjected to western blotting for analysis of nSMase1 phosphorylation.

### Co‐Immunoprecipitation

Following cell lysis with IP Lysis Buffer (Beyotime, P0013), protein concentration was determined by BCA assay. Lysates containing equal protein quantities were incubated with either primary antibody or species‐matched IgG control (1:100). Protein A/G magnetic beads (Thermo Fisher, 88802) were subsequently added and incubated for 2 h at 4 °C. The beads were then washed three times with ice‐cold IP lysis buffer, followed by resuspended in IP lysis buffer mixed with 5× loading buffer and denatured at 95 °C for 10 min.

For the detection of nSMase1 phosphorylation in vivo, ischemic ipsilateral hemispheres were dissected post‐MCAO and homogenized in 3 mL ice‐cold IP lysis buffer per hemisphere using a Miltenyi Biotec GentleMACS Dissociator. The homogenates were subsequently processed through the standardized co‐immunoprecipitation protocol as described above.

### Immunofluorescence Staining

Cells were seeded onto a confocal laser‐scanning microscope‐specific culture dish for the designated duration. Subsequently, the cells were fixed with 4% paraformaldehyde solution at room temperature. Permeabilization was achieved using 0.2% Triton X‐100. Primary antibodies diluted in antibody diluent with background reducing agents (DAKO, S3022) were added, followed by overnight incubation at 4 °C. The primary antibodies used including anti‐GFAP (CST, 3670), anti‐β‐III‐Tubulin (abcam, ab78078), anti‐nSMase (abcam, ab131330), anti‐mCherry (abcam, ab125096), anti‐HK2 (abcam, ab209847), anti‐LAMP1 (abcam, ab25630), anti‐Claudin 5 (Invitrogen, 35–2500). Fluorescently labeled secondary antibodies corresponding to the species of the primary antibodies were subsequently added. The secondary antibodies used including Donkey anti‐Rabbit IgG Alexa Fluor 647 (Invitrogen, A31573), Donkey anti‐Rabbit IgG Alexa Fluor 555 (Invitrogen, A31572), Donkey anti‐mouse IgG Alexa Fluor 488 (Invitrogen, A21202), Donkey anti‐mouse IgG Alexa Fluor 555 (Invitrogen, A31570). Cell nuclei were stained with Hoechst 33342 dye (Sigma Aldrich, 14533). To prevent the samples from drying, 200 µL of PBS was retained in the cell samples, followed by confocal imaging.

During tissue sectioning, the brain sections were routinely separated and fixed. Then, the samples were embedded in paraffin and cut into 5‐µm‐thick sections. After deparaffinization and hydration, the slices were subjected to antigen retrieval for 20 min and cooled to room temperature. The samples were incubated with the indicated primary antibodies overnight at 4 °C in an antibody diluent with background reducing reagents (DAKO). The samples were washed with PBS 3 times and incubated with the indicated fluorescence‐conjugated secondary antibodies for 1h. Then, the samples were washed and stained with Hoechst 33342 (5 µg mL^−1^) for 10 min. The film was sealed with a water‐soluble sealing agent. The slices were mounted and imaged by using a Nikon A1 Spectral Confocal Microscope (Nikon, Japan).

### siRNA, Plasmid Construction and Transfection

SiRNA was constructed and purchased from RiboBio Co., Ltd. (Guangzhou). Transfection was performed following the siRNA transfection reagent (MIKX, MK4018) instructions when the cells reached an appropriate density.

Plasmids were constructed from Tsingke biotechnology company. The method for plasmid extraction followed the instructions of the Tiangen High Purity Plasmid Extraction Kit (Tiangen, DP107‐02). The concentration of the plasmid was measured using a NanoDrop spectrophotometer. The transfection of the plasmid was conducted according to the protocol of the Lipofectamine 3000 Transfection Reagent Kit (Invitrogen, L3000‐15).

### AAV9 Construction and Infection

The adeno‐associated virus (pAAV9‐shortGFAP‐miR30 shHK2‐mCherry) used for specific infection and knockdown of astrocytes in vivo was constructed and packaged by OBiO Technology Co., Ltd. (Shanghai). This serotype of AAV9 has a short GFAP promoter for selectively knockdown HK2 in astrocytes. The miR30 system was used to construct a shRNA targeting HK2, which is fused with the mCherry fluorescent protein for viral tracing. The sequence of the interfering fragments is shown as below:

**Table:** 

Y6914 (shHK2)	GCATATGATCGCCTGCTTA
Y6915 (shHK2)	CCAGCTGTTTGACCACATT
Y6916 (shHK2)	GCTGCTGTTCCAAGGGAAA

### Brain Stereotactic Injection of AAV9 Virus

After inducing anesthesia in mice with isoflurane, the animals were fixed in a prone position on a stereotaxic apparatus, and anesthesia was maintained with a low dose of isoflurane. The mice were placed on a 37 °C heated surgical pad to maintain body temperature, ensuring unobstructed respiration. The skin over the skull was shaved, and an incision was made to expose the anterior and posterior fontanels. Using the stereotaxic apparatus, the coordinates were set to (anterior fontanel posterior −1.58 mm; lateral to midline +2.50 mm), and a blunt‐head drill was used to create a small hole in the skull. A microsyringe was advanced through the right side of the skull until it made contact with the cortical surface, with the *z*‐axis set to zero. The syringe was then lowered slowly to the coordinate −0.90 mm for injection. Using an electronic injection control system, 1 µL of virus was injected at a constant rate over 5 min. After the injection, the syringe was left in place for an additional 5 min before being slowly retracted. To prevent brain infection, bone wax was applied to the cranial hole following the injection. The skull was rinsed with saline, and the skin was sutured. Four weeks after viral infection, the corresponding MCAO model and subsequent procedures were initiated.

### Phos‐Tag SDS‐PAGE

The experimental methods followed the reagent instructions from APExBIO. During the preparation of protein samples, 100 µM MnCl_2_ should be added to chelate the EDTA in the samples. During the preparation of the separation gel, 40 µM Phos‐tag and 100 µM MnCl_2_ should be added. Electrophoresis was performed under constant current conditions. For transfer of gel‐separated proteins to PVDF membranes, gels were pretreated with washing in methanol‐free transfer buffer with 5 mM EDTA for 10 min twice to remove bivalent cations.

### TTC Staining

Brain tissues were frozen and sliced with a microtome into five coronal sections (2‐mm thick). The sections were then incubated in 1.0% 2,3,5‐triphenyltetrazolium chloride (TTC) (MP Biomedicals, 103126) at 37 °C for 20 min and fixed in a 4.0% paraformaldehyde solution overnight. After TTC staining, determination of infarct size was carried out by an independent investigator. For each coronal slice, the infarct tissue (unstained area) and total bihemispheric area were delineated in the scanned image with Adobe Photoshop CS6. The infarcted volume was calculated as the ratio of the total unstained areas (white) over the total bihemispheric area. During the whole procedure, investigators were blinded with the treatment group.

### Neurological Score

After 23 h of reperfusion, the Bederson's neurological score of mice was assessed the neurological function in a blinded manner. The neurologic findings were scored on a five‐point scale, a score of 0 indicates no detectable neurological deficit, a score of 1 indicates left foreleg inward and extension disorder when tail hoisted in the air, a score of 2 indicates contralateral circling, a score of 3 indicates falling toward the contralateral side, a score of 4 indicates no spontaneous locomotor activity, and 5 indicates dead. We excluded experimental mice with a score of 0 or 5, and mice with a neurological deficit score of 1–4 were included for subsequent analysis.

### Single‐Cell RNA Sequencing Data Analysis

The single‐cell RNA sequencing dataset analyzed in this study was obtained from the Gene Expression Omnibus (GEO) under accession number GSE174574. Standard single‐cell analysis workflows were applied. Raw count data were normalized, highly variable genes were identified, and the dataset was scaled. Principal component analysis (PCA) was used for dimensionality reduction, followed by unsupervised clustering and visualization with Uniform Manifold Approximation and Projection (UMAP).

Then we identified and filtered out predicted doublets using by DoubletFinder package, followed by removing low‐quality cells. Cells would be flagged as poor‐quality ones if they met one of the following thresholds: 1) the number of expressed genes was lower than 200 or larger than 7500; 2) 10% or more of UMIs were mapped to ribosomal genes; 3) 5% or more of UMIs were mapped to mitochondrial genes. Following quality control and batch effect correction, cells were re‐clustered to ensure consistent integration across samples.

Cell types were annotated based on canonical marker genes. Astrocytes were then extracted from the annotated dataset for downstream analysis. Differential gene expression analysis was performed on astrocytes between MCAO and sham groups. Gene Ontology (GO) enrichment analysis focused on the Cellular Component (CC) category was conducted using the differentially expressed genes. The most significantly enriched GO terms were visualized using bar plots.

### Statistical Analysis

For comparison of two groups, the two‐tailed independent Student's t‐test was used. For comparison of three or more groups, a one‐way analysis of variance (ANOVA) followed by Tukey's posthoc‐test. A *p*‐value of <0.05 was considered statistically significant. The statistical software was GraphPad Prism version 7.0.

## Conflict of Interest

The authors declare no conflict of interest.

## Author Contributions

C.C., Y.Z., and L.S. contributed equally to this work. C.C., W.Y., X.H., and J.D. performed conceptualization. C.C., L.S. and Y.Z. performed formal analysis. C.C., Y.Z., and J.W. performed data curation. X.H., W.Y., and J.D. performed project administration. C.C., Y.Z., L.S., F.M., Y.D., and S.H. performed the investigation. C.C. and L.S. performed the methodology. C.C. and Y.Z. wrote the original draft. W.Y., J.P., G.Y., and Y.H. Wrote, reviewed, and edited. W.Y., C.C., and Y. Z. performed funding acquisition. W.Y. and J.D. performed resources. W.Y. and X.H. performed supervision.

## Supporting information



Supporting Information

## Data Availability

The data that support the findings of this study are available from the corresponding author upon reasonable request.
