# Supplementary material for: Glycolytic Enzyme HK2 Phosphorylates nSMase1 to Promote Astrocytic Exosomes Biogenesis Contributing to Acute Ischemic Stroke Injury
Source: Adv Sci (Weinh). 2025 Jul 28;12(39):e01894. doi: 10.1002/advs.202501894 (PMC12533208; doi:10.1002/advs.202501894)
Supplement: Supplementary file 1 — Supporting Information [file ADVS-12-e01894-s001.docx]

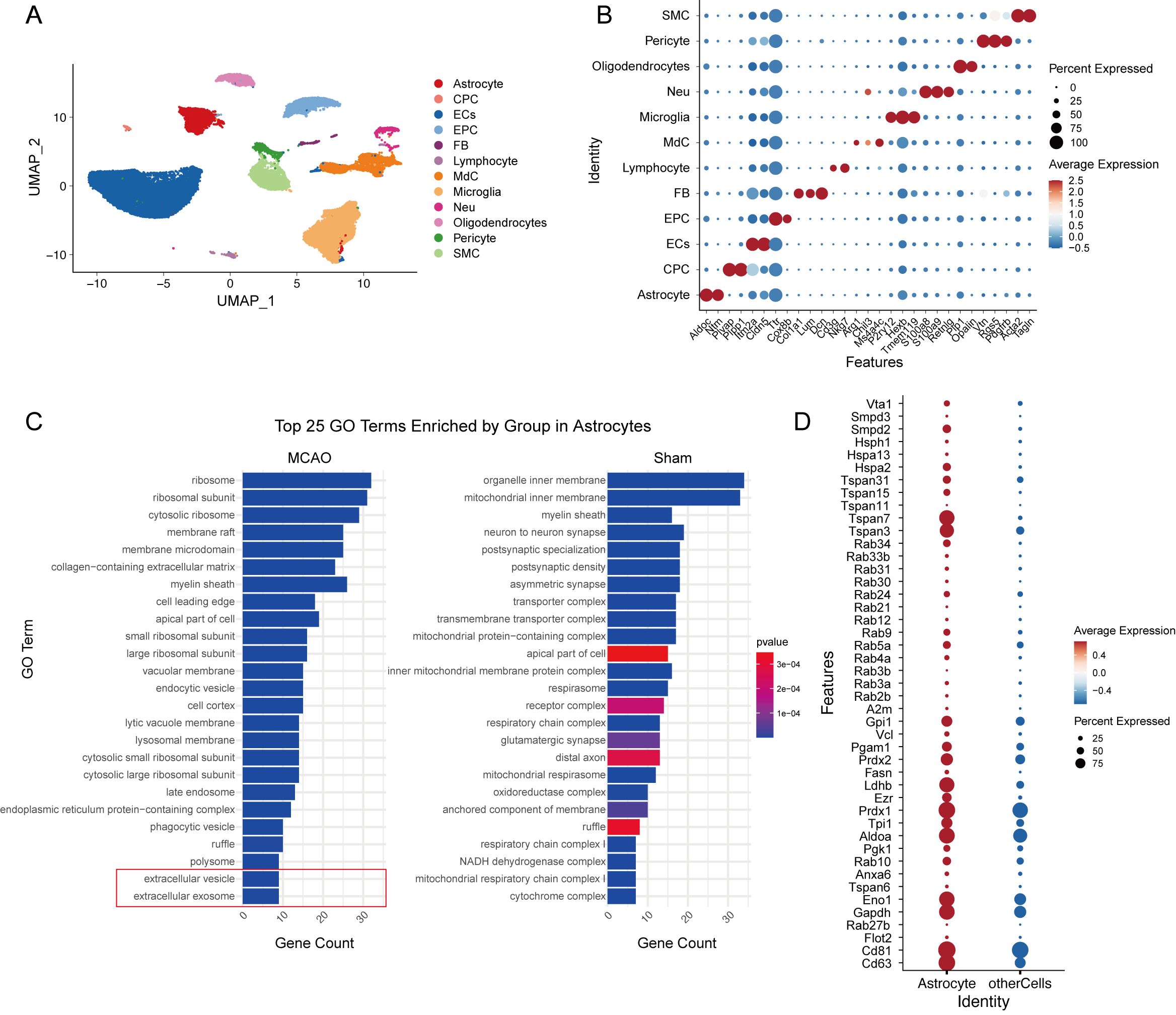


**Supplementary figure S1. Single-cell RNA sequencing data revealed that exosomes in the mouse brain after MCAO primarily originate from astrocytes**. **(A)** The UMAP plot visualizing clustering of single cells colored by annotated cell types. A total of 55,543 cells from the publicly available dataset GSE174574 are displayed, revealing 12 major cell types. **(B)** Bubble plot illustrating the expression of canonical marker genes across cell types. Dot size represents the proportion of cells expressing each gene, and color indicates normalized expression levels. **(C)** Go terms enriched in astrocytes from MCAO and sham groups. **(D)** Bubble plot displaying the expression of exosomal protein marker genes in astrocytes and other cells from MCAO and sham groups. Dot size represents the fraction of expressing cells, and color reflects normalized expression levels. CPC, choroid plexus epithelial cells; EC, endothelial cells; EPC, ependymocytes; FB, perivascular fibroblast-like cells; MdC, monocyte-derived cells; Neu, neutrophils; SMC, vascular smooth muscle cells.


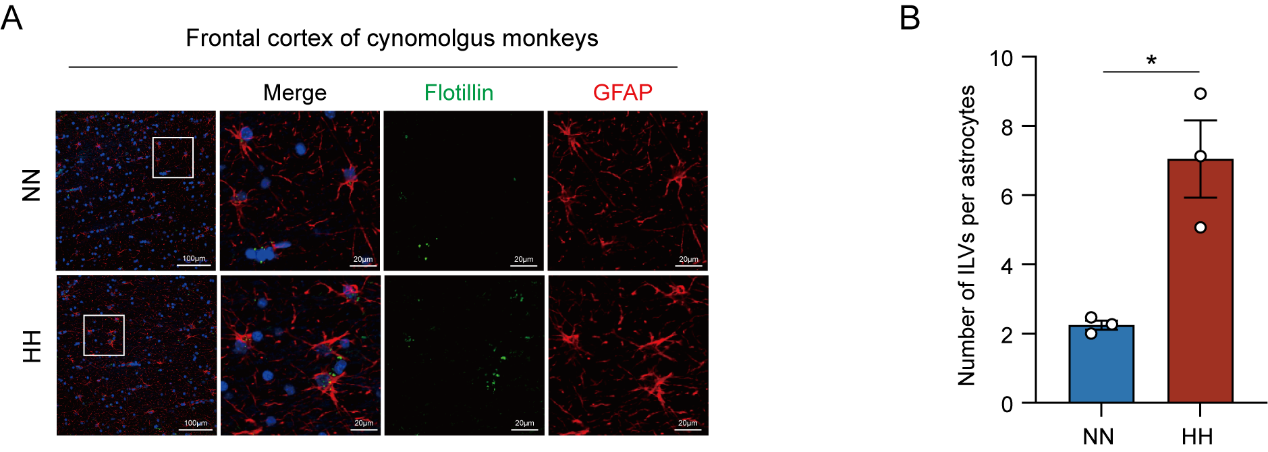


**Supplementary figure S2. (A)** Representative images of Flot^+^ ILVs (green) in astrocytes (red) in frontal cortex of crab-eating macaques treated with or without acute hypobaric hypoxia treatment. bar=100μm (left), bar=20μm (right) **(B)** Quantity statistics of Flot^+^ ILVs in (A). HH, hypobaric hypoxia. NN, normobaric normoxia. Statistical analysis was performed by unpaired Student's t test. *, p<0.05.
